# Avian influenza A viruses exhibit plasticity in sialylglycoconjugate receptor usage in human lung cells

**DOI:** 10.1128/jvi.00906-23

**Published:** 2023-10-16

**Authors:** Chieh-Yu Liang, Iris Huang, Julianna Han, Boopathi Sownthirarajan, Katarina Kulhankova, Nathan B. Murray, Mehrnoush Taherzadeh, Stephanie Archer-Hartmann, Lauren Pepi, Senthamizharasi Manivasagam, Jesse Plung, Miranda Sturtz, Yolanda Yu, Olivia A. Vogel, Matheswaran Kandasamy, Francoise A. Gourronc, Aloysius J. Klingelhutz, Biswa Choudhury, Lijun Rong, Jasmine T. Perez, Parastoo Azadi, Paul B. McCray, Sriram Neelamegham, Balaji Manicassamy

**Affiliations:** 1 Department of Microbiology and Immunology, University of Iowa, Iowa City, lowa, USA; 2 Department of Microbiology, University of Chicago, Chicago, Illinois, USA; 3 Department of Pediatrics, University of Iowa, Iowa City, lowa, USA; 4 Complex Carbohydrate Research Center, University of Georgia, Athens, Georgia, USA; 5 Department of Cellular and Molecular Medicine, University of California San Diego, La Jolla, California, USA; 6 Department of Microbiology and Immunology, University of Illinois, Chicago, Illinois, USA; 7 Department of Chemical and Biomedical Engineering, University at Buffalo, Buffalo, New York, USA; The Peter Doherty Institute for Infection and Immunity, Melbourne, Victoria, Australia

**Keywords:** avian influenza viruses, sialic acid, virus entry, viral receptor, glycosylation

## Abstract

Influenza A viruses (IAV) utilize sialic acid (Sia) containing cell surface glycoconjugates for host cell infection, and IAV strains from different host species show preferences for structurally distinct Sia at the termini of glycoconjugates. Various types of cell surface glycoconjugates (N-glycans, O-glycans, glycolipids) display significant diversity in both structure and carbohydrate composition. To define the types of sialylglycoconjugates that facilitate IAV infection, we utilized the CRISPR/Cas9 technique to truncate the three major types of glycoconjugates, either individually or in combination, by targeting glycosyltransferases essential to glycan biosynthesis in a human lung epithelial cell line. Our studies show that both human and avian IAV strains do not display strict preferences for a specific type of glycoconjugate. Interestingly, truncation of the three major types of glycoconjugates significantly decreased replication of human IAV strains, yet did not impact replication of avian IAV strains. Unlike human IAV strains, avian IAV strains were able to efficiently utilize other less prevalent shorter glycoconjugates such as sialyl Tn and sialyl T antigens. Taken together, our studies demonstrate that avian IAV strains utilize a broader repertoire of glycoconjugates for host cell infection as compared to human IAV strains.

**IMPORTANCE:**

It is well known that influenza A viruses (IAV) initiate host cell infection by binding to sialic acid, a sugar molecule present at the ends of various sugar chains called glycoconjugates. These sugar chains can vary in chain length, structure, and composition. However, it remains unknown if IAV strains preferentially bind to sialic acid on specific glycoconjugate type(s) for host cell infection. Here, we utilized CRISPR gene editing to abolish sialic acid on different glycoconjugate types in human lung cells, and evaluated human versus avian IAV infections. Our studies show that both human and avian IAV strains can infect human lung cells by utilizing any of the three major sialic acid-containing glycoconjugate types, specifically N-glycans, O-glycans, and glycolipids. Interestingly, simultaneous elimination of sialic acid on all three major glycoconjugate types in human lung cells dramatically decreased human IAV infection, yet had little effect on avian IAV infection. These studies show that avian IAV strains effectively utilize other less prevalent glycoconjugates for infection, whereas human IAV strains rely on a limited repertoire of glycoconjugate types. The remarkable ability of avian IAV strains to utilize diverse glycoconjugate types may allow for easy transmission into new host species.

## INTRODUCTION

Host glycans expressed on the cell surface in the form of glycoconjugates (glycoproteins, glycolipids, and glycosaminoglycans) serve as the main entry receptor(s) for a variety of viruses (1, 2). This is exemplified by influenza A viruses (IAV), which utilize sialic acid (Sia) as the host entry receptor. Sia belongs to a family of 9-carbon sugars (>90 members) predominantly present at the termini of cell surface glycoconjugates in the deuterostome lineage, including vertebrates (3, 4). As such, IAV can infect a broad range of species, including humans, aquatic birds, domestic birds, swine, and sea mammals, with aquatic birds serving as the reservoir species for almost all subtypes of IAV (5, 6). In humans and swine, IAV infections occur in the upper respiratory tract, whereas IAV infections predominantly occur in the gastrointestinal tract of avian species. IAV strains from various host species show preferences for distinct modifications on Sia molecules in the context of their linkages and backbone sugar chains (7). Human IAV strains preferentially bind to Sia linked to the penultimate galactose via a α2,6 carbon linkage (i.e., Siaα2–6Galβ), whereas avian strains prefer α2,3-linked Sia moieties (8, 9). Differences in IAV host tissue tropism have been attributed to the availability of different Sia types, as Siaα2–6Gal is abundant in the human upper respiratory tract and Siaα2–3Gal is highly expressed in the avian intestinal tract (10). As both types of sialoglycans are expressed in the respiratory tract of swine, they are able to support the replication of both avian and human IAV strains (10). In the past 100 years, IAV strains from zoonotic reservoirs have crossed the species barrier and caused four pandemics in humans. These pandemic strains demonstrate the unique ability to bind to Sia receptors present in both human and avian hosts (8, 11). Thus, the ability to bind Siaα2–6Gal versus Siaα2–3Gal is considered a critical factor in determining IAV host range (8).

Sia containing glycoconjugates are attached to proteins through asparagine (N-glycan) or serine/threonine residues (O-glycan), or to glycosphingolipids (GSL). IAV entry is initiated by binding of viral hemagglutinin (HA) to cell surface sialoglycans, which triggers intracellular signaling cascades, such as receptor tyrosine kinases, that facilitate virion uptake and fusion (12). Prior studies suggest that HA can engage Sia modifications present on several cell surface proteins, such as epidermal growth factor receptor, calcium-dependent voltage channel (Ca_v_1.2i), natural killer cell receptors (NKP44/46), and nucleolin, to facilitate virion uptake (7). Moreover, in the absence of Sia, some C-type lectins predominantly expressed on antigen presenting cells, such as DC-SIGN, L-SIGN, mannose receptor, etc., can also facilitate IAV uptake by binding to glycan moieties on viral glycoproteins (7). The glycoconjugate structural features necessary for HA binding have been identified using chemically defined glycan arrays [Consortium for Functional Glycomics (CFG)] and shotgun lung tissue glycan arrays (11, 13 – 17). These studies suggest that human IAV strains preferentially bound to long branched sialoglycans with poly-lactosamine (polyLN) repeats that had an “umbrella-like” topology, whereas avian IAV strains preferentially bound to short sialoglycans with a single lactosamine that had a “cone-like” topology, indicating that glycan topology can also determine host range (18). In agreement, circulating human H3N2 viruses have evolved to utilize extended branched polyLN glycans (N-glycans), while the parental pandemic H3N2 strain preferentially bound to short sialyl-LN glycans (19 – 21). In array slides, glycans are immobilized in non-natural configurations at a high density with uniformity and hence, the conclusions can be biased on the repertoire of glycans presented (8, 9).

Two prior studies using Chinese hamster ovary (CHO) cell lines lacking N-glycans (due to mutations in an essential biosynthesis gene Mgat1) arrived at opposite conclusions—N-glycans were absolutely required for IAV infection (22), and N-glycans were not an absolute requirement (23). The results from the latter study are consistent with binding studies performed using recombinant HA and a panel of human embryonic kidney (HEK) 293 CRISPR knockout cells (24). In addition, studies in HEK293 CRISPR KO cells also reconfirmed the Siaα2,3 versus Siaα2,6 binding preferences for avian and human HAs, respectively (24). However, these studies were limited to the assessment of HA binding; IAV infection and replication were not evaluated. Importantly, as HEK293 cells likely do not mimic the glycan repertoire of human lung epithelial cells, our understanding of the types of glycoconjugates utilized by human and avian IAV strains for infection of human lung cells remains incomplete.

In this study, we utilized human lung epithelial cell lines (A549, Nuli-1) as well as primary human airway epithelial cells to assess the preferences for different glycoconjugate types by avian versus human IAV strains in the context of infection. To this end, a comprehensive panel of CRISPR gene edited A549 cells were generated that contained truncated N-glycans (N)^−^, O-glycans (O)^−^, or glycosphingolipids (G)^−^, either individually or in combination, by disrupting the expression of glycosyltransferases *MGAT1*, *C1GALT1*, or *UGCG*, respectively. Surprisingly, truncation of individual glycans [(N)^−^, (O)^−^, (G)^−^] in A549 cells had no effect on the replication of multiple IAV strains; concurrent truncation of two glycan types [(NO)^−^, (NG)^−^] in A549 cells showed a modest decrease in H1N1 infection, yet no defect in avian H5N1 infection. Importantly, concurrent truncation of all three glycan types [(NOG)^−^] in A549 cells resulted in a 1–3 log decrease in viral titers for human H1N1 and H3N2 viruses, yet showed little to no change in titers for several avian IAV strains (H5N1, H7N7, H7N9, H9N2, etc.). The robust replication of avian IAV strains observed in A549 [(NOG)^−^] cells was dependent on Sia receptors, likely through shorter glycoconjugates such as sialyl Tn and sialyl T antigens. We confirmed these findings in primary human airway CRISPR KO cells with truncated N- or O-glycans. Thus, beyond the known Siaα2,3 versus Siaα2,6 differences, glycoconjugate types also determine the functional diversity of IAV strains. Taken together, our study is the first to demonstrate the differences in glycoconjugate preferences for avian versus human IAV strains. Importantly, our studies show that avian IAV strains utilize structurally diverse glycoconjugates for host cell infection as compared to human IAV strains.

## RESULTS

### Generation of A549 cells lacking Sia on N- or O-glycans

**Fig 1:**
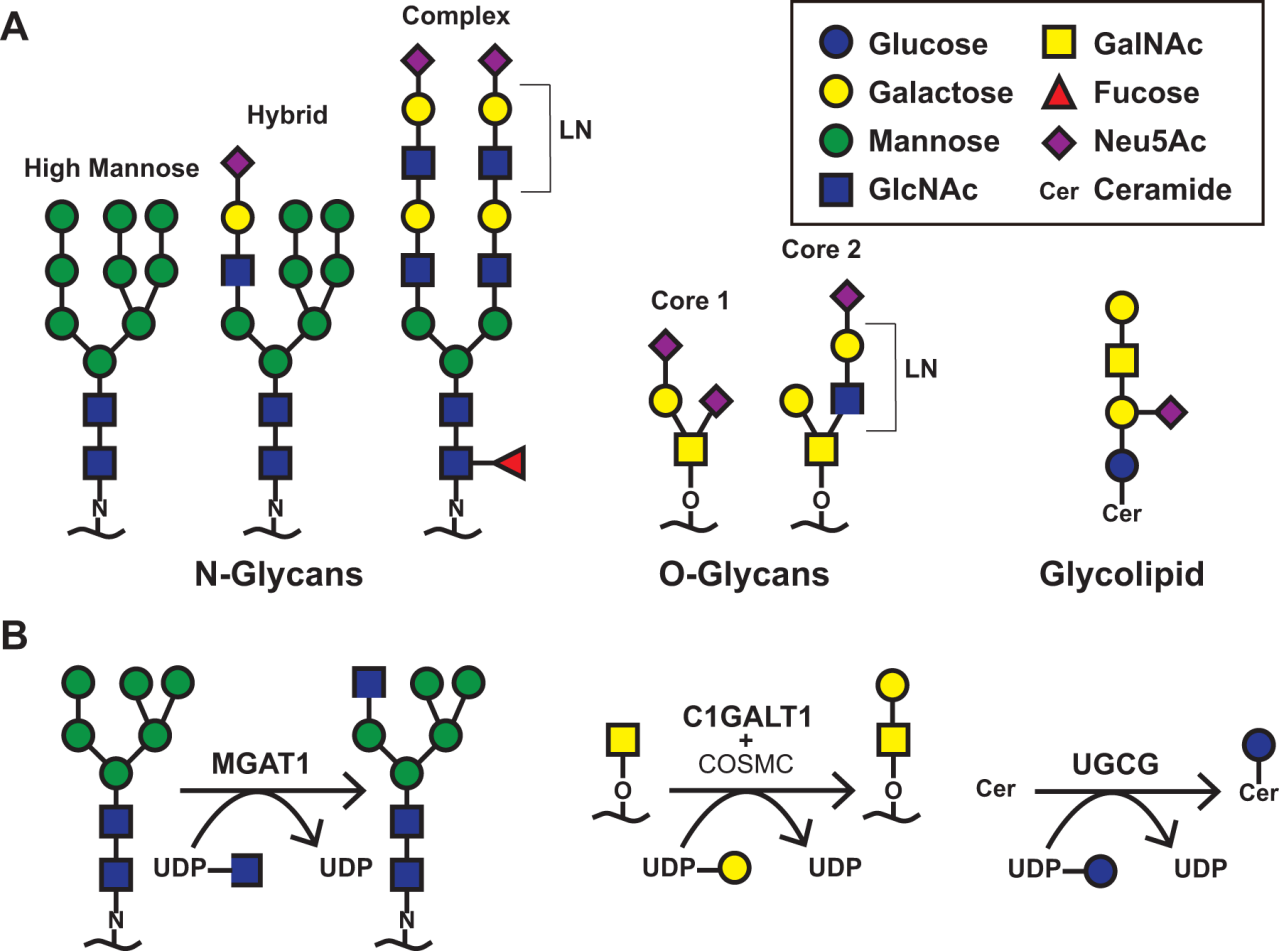
Representative structures of Sia-containing glycoconjugates and the key glycosyltransferases essential for glycoconjugate biosynthesis. (**A**) Schematic representation of different glycoconjugates terminating with Sia. For N-glycans: high-mannose, hybrid, and complex structures are shown; for O-glycans: Core 1 and Core 2 structures are shown; ganglioside GM1 type glycolipid is shown. (**B**) Key glycosyltransferases essential for biosynthesis of individual glycoconjugate types. MGAT1 is necessary for the formation of hybrid and complex N-glycans. C1GALT1 is essential for the synthesis of Core 1 and Core 2 O-glycans. UGCG is essential for the first step of glycolipid biosynthesis.

We ablated terminal Sia modifications on specific glycoconjugate types in human lung epithelial A549 cells by targeting glycosyltransferases that are essential for the elongation of N- or O-linked glycans (Fig. 1A) (25). Here, using the CRISPR/Cas9 technology, we targeted the glycosyltransferases *MGAT1* or *C1GALT1* to generate A549 cells with truncated N-glycans [(N)^−^] or O-glycans [(O)^−^], respectively (Fig. 1B; Table S1). Loss of individual glycosyltransferases was confirmed by Sanger sequencing of the sgRNA target site, Western blot analysis, and lectin staining with *Sambucus Nigra* lectin (SNA; specific for α2,6-linked Sia), *Vicia Villosa* lectin (VVL, specific for terminal GalNAc), and Cholera toxin B subunit (CTB, specific for GM1 gangliosides) (Table S2; Fig. S1). As previously described, we observed decreased SNA binding in *MGAT1* KO cells [(N)^−^] as compared to wild type (WT) A549 cells, demonstrating that truncation of N-glycan structures resulted in the loss of N-glycans with α2,6-linked Sia moieties (Fig. S1C) (25). As expected, we observed similar levels of VVL and CTB binding in *MGAT1* KO cells [(N)^−^] as compared to WT A549 cells, indicating that the expression of O- and GSL-glycans was not significantly altered upon loss of MGAT1. In *C1GALT1* KO cells [(O)^−^], we observed increased VVL binding due to higher levels of unmodified GalNAc on truncated O-glycans (Fig. S1C). As expected, *C1GALT1* KO cells [(O)^−^] showed similar levels of SNA and CTB binding as compared to WT A549 cells, indicating that the expression of N- and GSL-glycans remained unaltered. Taken together, we successfully generated A549 cells lacking Sia specifically on N- or O-glycans.

### Sia-containing N-glycans or O-glycans are not essential for IAV replication

**Fig 2:**
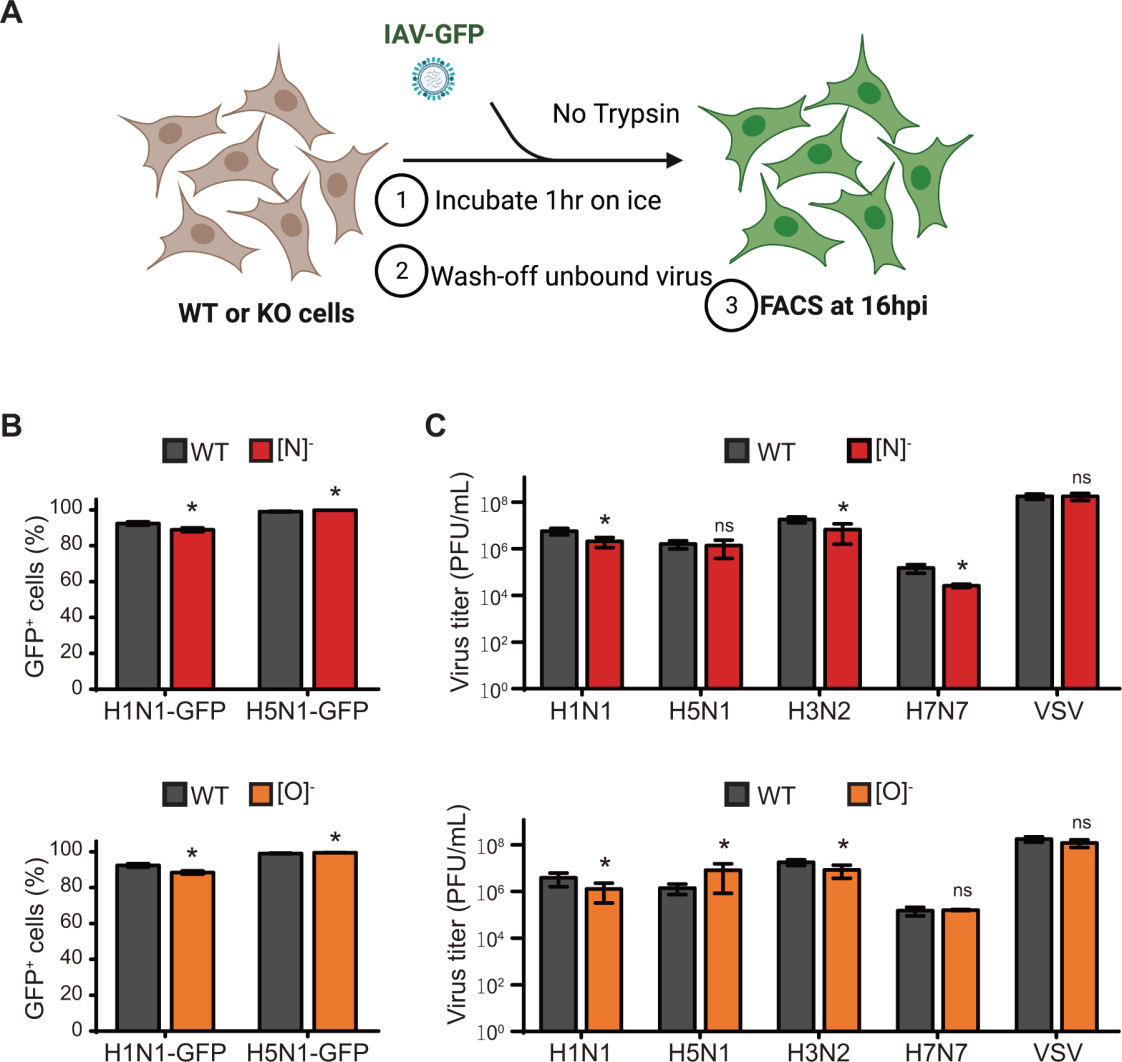
Truncation of N- or O-glycans individually does not affect IAV infection or replication. (**A**) Experimental outline for single-cycle infection with IAV-GFP. WT and KO A549 cells seeded in 6-well dishes were infected with H1N1-GFP or H5N1-GFP at a high MOI (MOI = 3) without TPCK-treated trypsin, and at 16 hpi, the percentage (%) of GFP expressing cells was determined by flow cytometric analysis. (**B**) Single-cycle infection assays with H1N1-GFP and H5N1-GFP in individual glycan KO A549 cells. Top: comparison of infection levels between WT and [N]^-^ KO cells; bottom: comparison of infection levels between WT and [O]^-^ KO cells. Data are represented as mean percentage of GFP+ cells from triplicate samples ± SD. (**C**) Multi-cycle replication assays with various IAV strains and VSV in individual glycan KO A549 cells. WT and KO cells seeded in 6-well dishes were infected with various IAV strains or VSV at a low MOI (MOI = 0.001–0.01) in the presence of TPCK-treated trypsin, and at 48 hpi, viral titers in the supernatants were determined by plaque assay in MDCK cells. Top: comparison of viral replication in WT and [N]^-^ KO A549 cells; bottom: comparison of viral replication in WT and [O]^-^ KO A549 cells. Data are represented as mean titer of triplicate samples ± SD (PFU/mL). *Denotes *P*-value ≤ 0.05. ns is non-significant. Data are representative of at least three independent experiments.

To determine if removal of terminal Sia on either N- or O-glycans impaired IAV infection, we performed single-cycle infection assays in (N)^−^ and (O)^−^ KO A549 cells with H1N1 (H1N1-GFP) and H5N1 (H5N1-GFP) viruses at a high multiplicity of infection (MOI) (MOI = 3), and assessed GFP expression at 16 hours post-infection (hpi) by flow cytometry (Fig. 2A and B). We observed modest differences in the percentage of GFP positive cells between infected WT A549 cells and (N)^−^ or (O)^−^ KO cells, indicating that truncation of N- or O-glycans individually did not grossly affect single-cycle IAV infection (Fig. 2B). In addition, the mean fluorescent intensity (MFI) of GFP was similar between WT A549 and KO cells, indicating equivalent levels of replication in all three cell types (data not shown). Next, we performed multi-cycle replication assays in (N)^−^ and (O)^−^ KO cells with four different IAV strains at a low MOI (MOI = 0.01–0.001) and observed robust replication for all four IAV strains in both (N)^−^ and (O)^−^ KO cells at levels similar to WT A549 cells (Fig. 2C). Similarly, the replication of vesicular stomatitis virus (VSV), which enters host cells through interactions with the low-density lipoprotein-receptor and thus would be unimpaired by truncation of sialoglycans, also remained unaffected in these KO cells (26). Taken together, these results demonstrate that loss of Sia on N- or O-glycans did not affect IAV replication, indicating that they are not solely essential for IAV entry.

### Sia-containing O-glycans or glycosphingolipids can individually support robust IAV replication

Next, we generated A549 double knockout (DKO) cells with truncations in both N- and O-glycans [(NO)^−^ DKO; expressing only glycosphingolipids (GSL)] as well as DKO cells with truncations in both N-glycans and GSL [(NG)^−^ DKO; expressing only O-glycans], and confirmed truncation of the intended glycans as described above (Fig. S2A and B; Table S2). Along with the previously described lectins (SNA, VVL, and CTB), we also assessed binding of *Galanthus Nivalis* lectin (GNL), which shows affinity for high mannose containing N-glycans. We observed increased GNL binding in both (NO)^−^ and (NG)^−^ DKO cells as compared to WT A549 cells, as loss of MGAT1 increases the levels of high mannose containing N-glycans (Fig. S2B) (25). In addition, we confirmed the lack of SNA binding in both (NO)^−^ and (NG)^−^ DKO cells. The disruption of O-glycans or GSL structures in (NO)^−^ and (NG)^−^ DKO cells was verified by increased VVL binding and decreased CTB binding, respectively.

**Fig 3:**
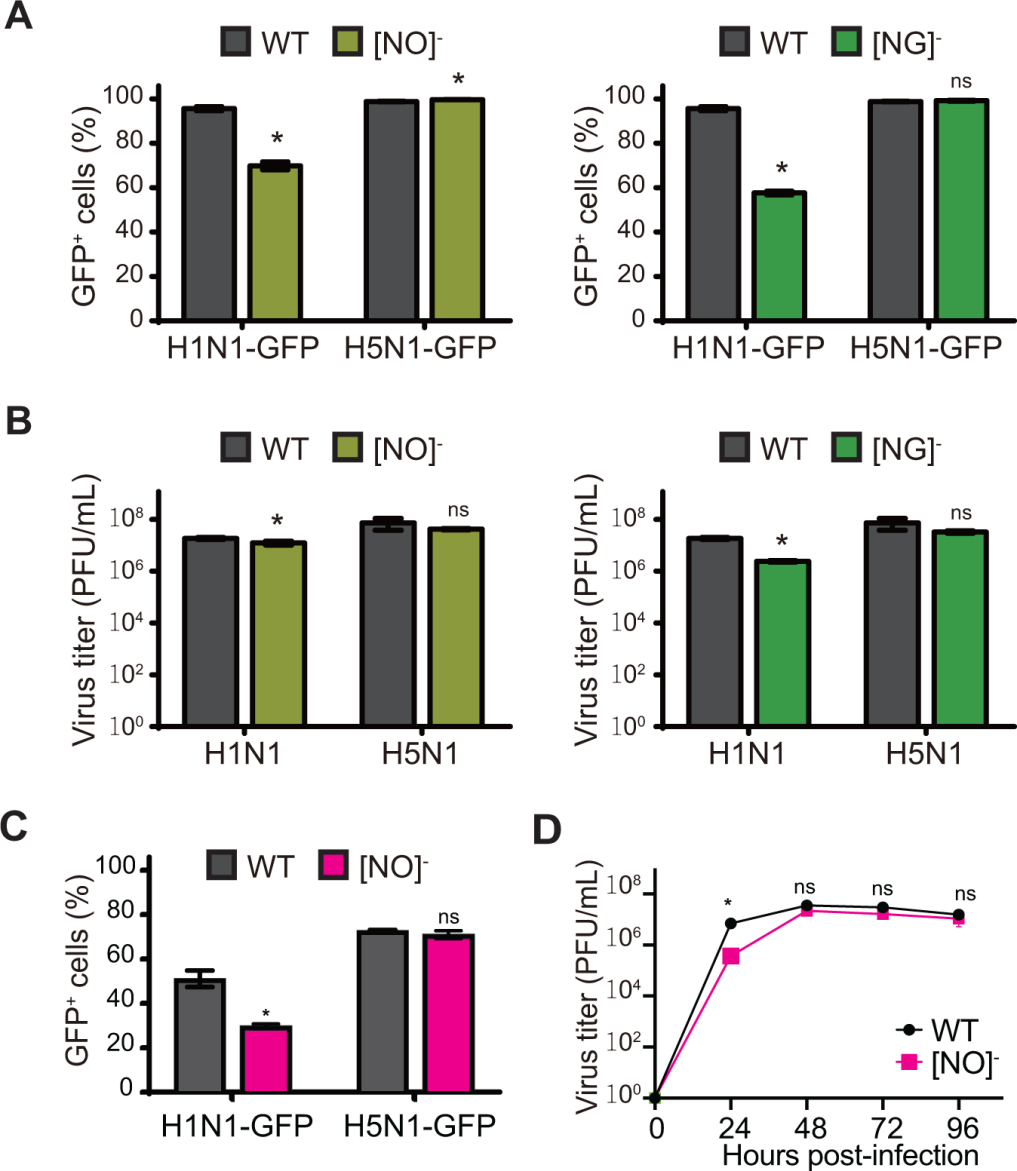
Concurrent truncation of two glycan types decreases H1N1 but not H5N1 infection. (**A**) Single-cycle infection assays with H1N1-GFP and H5N1-GFP in [NO]^-^ and [NG]^-^ DKO A549 cells. WT A549 and DKO cells seeded in 6-well dishes were infected with H1N1-GFP or H5N1-GFP at a high MOI (MOI = 3) without TPCK-treated trypsin, and at 16 hpi, the % of GFP expressing cells was determined by flow cytometric analysis. Left: comparison of infection levels between WT A549 and [NO]^-^ DKO cells; right: comparison of infection levels between WT A549 and [NG]^-^ DKO cells. Data are presented as mean percentage of GFP+ cells from triplicate samples ± SD. (**B**) Multi-cycle replication assays with H1N1 and H5N1 viruses in [NO]^-^ and [NG]^-^ DKO cells. WT A549 and DKO cells seeded in 6-well dishes were infected at a low MOI with H1N1 (MOI = 0.01) or H5N1 (MOI = 0.001) in the presence of TPCK-treated trypsin and at 48 hpi, viral titers in the supernatants were determined by plaque assay in MDCK cells. Left: comparison of viral replication in WT and [NO]^-^ DKO cells; right: comparison of viral replication in WT and [NG]^-^ DKO cells. (**C**) Single-cycle infection assays with H1N1-GFP and H5N1-GFP in [NO]^-^ DKO Nuli-1 cells. WT and DKO Nuli-1 cells seeded in 6-well dishes were infected with H1N1-GFP or H5N1-GFP at a high MOI (MOI = 3) without TPCK-treated trypsin and at 16 hpi, the % of GFP positive cells was determined by flow cytometric analysis. Data are represented as mean percentage of GFP+ cells from triplicate samples ± SD. (**D**) Multi-cycle replication assays with H5N1 virus in [NO]^-^ DKO Nuli-1 cells. WT and DKO Nuli-1 cells seeded in 6-well dishes were infected at a low MOI with H5N1 (MOI = 0.001) in the presence of TPCK-treated trypsin, and viral titers in the supernatants at different hpi were determined by plaque assay in MDCK cells. Data are represented as mean titer of triplicate samples ± SD (PFU/mL). *Denotes *P*-value ≤ 0.05. ns is non-significant. Data are representative of at least three independent experiments.

To determine if concurrent truncation of two glycan types impairs IAV infection, we performed single-cycle infections in (NO)^−^ and (NG)^−^ DKO cells with H1N1-GFP or H5N1-GFP. We observed a 30%–40% decrease in H1N1-GFP infection for both (NO)^−^ and (NG)^−^ DKO cells as compared to WT A549 cells (Fig. 3A); in contrast, H5N1-GFP infection remained high in both DKO cell types at levels comparable to WT A549 cells. Next, we performed multi-cycle replication assays in (NO)^−^ and (NG)^−^ DKO cells with H1N1 or H5N1 viruses (Fig. 3B; Fig. S2C). Surprisingly, replication of both IAV strains remained high in (NO)^−^ and (NG)^−^ DKO cells, with peak viral titers reaching up to ~10^7^ PFU/mL at 48 hpi. These results demonstrate that Sia on a single major glycoconjugate type (O-glycans or GSL) is sufficient to support robust replication of H1N1 and H5N1, albeit with reduced H1N1-GFP infection.

### Validation of IAV glycoconjugate requirements in DKO Nuli-1 cells

To validate our findings in another human lung cell line, we investigated the importance of different glycoconjugate types for IAV replication in Nuli-1 cells, which is an immortalized cell line (27). We generated DKO Nuli-1 cells with concurrent truncation of both N- and O-glycans by targeting the *MGAT1* and *C1GALT1* genes via CRISPR editing and confirmed loss of the intended glycans as described above [(NO)^−^ DKO Nuli-1 cells; Fig S2D]. Similar to our findings in (NO)^−^ DKO A549 cells, we observed reduced H1N1-GFP infection in (NO)^−^ DKO Nuli-1cells as compared to control WT Nuli-1 cells (Fig. 3C). In contrast, H5N1-GFP infection in (NO)^−^ DKO Nuli-1 cells remained high, with levels similar to control WT Nuli-1 cells. In addition, in multi-cycle replication assays, H5N1 replication in (NO)^−^ DKO Nuli-1 cells was comparable to control WT Nuli-1 cells (Fig. 3D). Unlike H5N1, H1N1 showed poor replication in control WT Nuli-1 cells and hence, we did not perform multi-cycle replication assays with H1N1. Taken together, our studies in (NO)^−^ DKO Nuli-1 cells confirmed our findings in A549 cells that Sia on GSL is sufficient for robust H5N1 replication.

### Generation and characterization of A549 cells lacking Sia on three major glycoconjugate types

**Fig 4:**
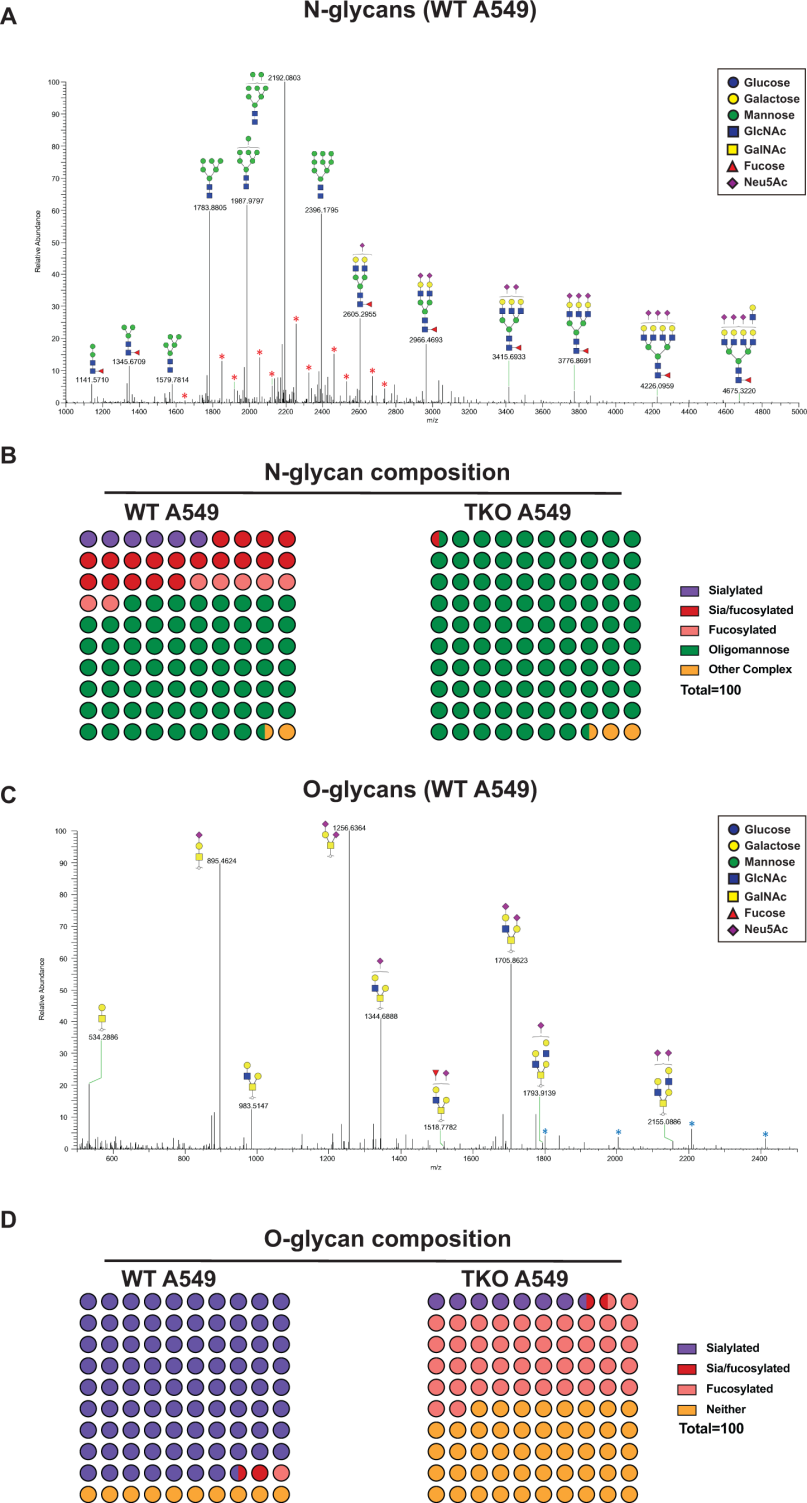
Glycomic profiling of N- and O-glycan structures in WT and [NOG]^-^ TKO A549 cells. N-glycans and O-glycans were extracted from uninfected WT and [NOG]^-^ TKO A549 cells and subjected to nESI-MS/MS analysis. (**A**) ESI-MS spectrum of N-glycans in WT A549 cells. Red asterisks indicate (M + NaCHO_2_) or (M + 2NaCHO_2_) adduct of an already displayed glycoform. (**B**) Comparison of N-glycans with different terminating residues expressed in WT and [NOG]^-^ TKO A549 cells. (**C**) ESI-MS spectrum of O-glycans in WT A549 cells. Blue asterisks indicate reduced N-glycan mass. (**D**) Comparison of O-glycans with different terminating residues expressed in WT and [NOG]^-^ TKO A549 cells.

Next, we generated triple KO (TKO) A549 cells with truncations in N-glycans, O-glycans, and GSL [(NOG)^−^ TKO] and confirmed loss of the intended glycan types as described above (Fig. S3A and B; Table S2). As anticipated, (NOG)^−^ TKO A549 cells showed increased GNL binding, decreased SNA binding, increased VVL binding, and decreased CTB binding (Fig. S3B). In addition, Sanger sequencing of the sgRNA target region confirmed the loss of UGCG in (NOG)^−^ TKO cells (Table S2). To define the structural features of N- and O-glycans expressed in (NOG)^−^ TKO cells versus WT A549 cells, we performed glycomic profiling by nano-electrospray ionization tandem mass spectrometry (nESI-MS/MS) (Fig. 4; Fig. S4). In WT A549 cells, we detected bi-, tri-, and tetra-antennary N-glycan structures with LN units terminating with Sia as well as N-glycan precursors terminating in mannose (Fig. 4A). In contrast, as anticipated, nearly all of the detected N-glycan structures terminated in high mannose residues (>95%) in (NOG)^−^ TKO cells (Fig. S4A and 4B). We did detect a very minor portion of Sia-containing N-glycan (~0.3%) in (NOG)^−^ TKO cells but are unsure of their origin. In our O-glycan profiling of WT A549 cells, we predominantly observed Core 1 and Core 2 structures, with the majority of the O-glycans containing single lactosamine (LN) and terminating in sialic acid (~90%) (Fig. 4C and D); in contrast and as anticipated, we did not observe Core 1 and Core 2 O-glycan structures in (NOG)^−^ TKO cells (Fig. S4B); however, we detected other remaining O-glycan Cores such as sialyl Tn antigen and Sialyl T antigens, with ~7% of these O-glycans terminating in Sia (Fig. 4D). To confirm the expression of STn in (NOG)^−^ TKO cells, we performed flow cytometric analysis with an STn antigen specific antibody and observed increased STn expression in (NOG)^−^ TKO cells as compared to WT A549 cells (Fig. S5). Our assessment of GSLs showed complete absence of glucosylceramide precursors in (NOG)^−^ TKO cells as compared to WT A549 cells (data not shown); however, due to similarities in the molecular masses of galactosylceramides and glucosylceramides, we were unable to definitively confirm the loss of glucosylceramide containing GSL in (NOG)^−^ TKO cells. Together, these results confirmed truncation of these three major glycoconjugates in (NOG)^−^ TKO A549 cells and importantly, (NOG)^−^ TKO cells completely lacked LN unit containing N- and O-glycans.

### H1N1 requires Sia on one of the three major glycoconjugates for robust replication

**Fig 5:**
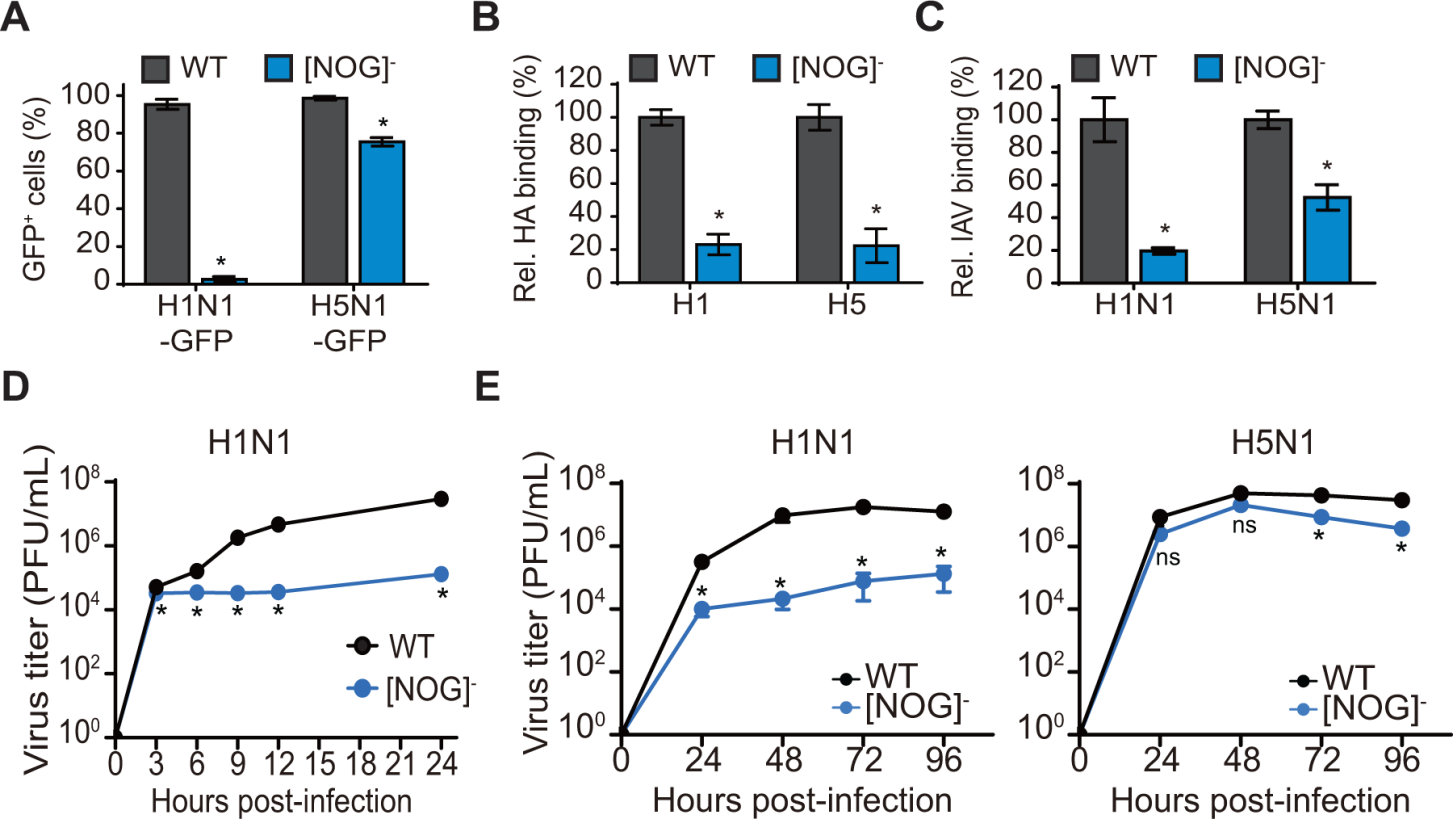
H5N1 but not H1N1 shows robust infection and replication in [NOG]^-^ TKO A549 cells. (**A**) Single-cycle infection assays with H1N1-GFP and H5N1-GFP in [NOG]^-^ TKO cells. WT and TKO A549 cells seeded in 6-well dishes were infected with H1N1-GFP or H5N1-GFP at a high MOI (MOI = 3) without TPCK-treated trypsin, and at 16 hpi, the % of GFP positive cells was determined by flow cytometric analysis. Data are represented as mean % of GFP+ cells from triplicate samples ± SD. (**B**) Comparison of cell surface binding of purified HA in [NOG]^-^ TKO A549 cells. WT or [NOG]^-^ TKO A549 cells were incubated with purified H1 or H5 subtype HA on ice, and HA binding was measured by flow cytometry. (**C**) Comparison of cell surface binding of H1N1 and H5N1 virions in [NOG]^-^ TKO A549 cells. WT or [NOG]^-^ TKO A549 cells were incubated with H1N1 or H5N1 virus (MOI = 100) on ice, and virion binding was measured by flow cytometry. For B and C, data are represented as mean relative binding from triplicate samples ± SD. (**D**) Single-cycle replication assays with H1N1 in [NOG]^-^ TKO A549 cells. WT and TKO A549 cells seeded in 6-well dishes were infected at an MOI = 3 without TPCK-treated trypsin, and at various times post-infection, supernatants were collected and viral titers were determined after the addition of TPCK-treated trypsin 1 hour prior to plaque assay. (**E**) Multi-cycle replication assays with H1N1 and H5N1 in [NOG]^-^ TKO A549 cells. WT and TKO A549 cells seeded in 6-well dishes were infected at a low MOI with H1N1 (MOI = 0.01) or H5N1 (MOI = 0.001) in the presence of TPCK-treated trypsin, and viral titers in the supernatants at different hpi were determined by plaque assay in MDCK cells. For D and E, data are represented as mean titer of triplicate samples ± SD (PFU/mL). *Denotes *P*-value ≤ 0.05. ns is non-significant. Data are representative of at least three independent experiments.

Next, we compared single-cycle infections of H1N1-GFP and H5N1-GFP in (NOG)^−^ TKO and WT A549 cells. H1N1-GFP infection was drastically reduced by >95% in (NOG)^−^ TKO cells as compared to WT A549 cells (Fig. 5A). Surprisingly, we only observed an ~20% reduction in H5N1-GFP infection in (NOG)^−^ TKO cells as compared to WT A549 cells. To confirm that the observed reduction in IAV infection was due to decreased virus binding, we performed cell surface binding assays with purified HA proteins and IAV virions. In HA binding assays, both H1 and H5 HA proteins showed reduced binding in (NOG)^−^ TKO cells as compared to WT A549 cells (Fig. 5B). Interestingly, H5N1 virions showed higher cell surface binding as compared to H1N1 virions in (NOG)^−^ TKO cells (Fig. 5C); however, the levels of binding for both H1N1 and H5N1 virions were lower in (NOG)^−^ TKO cells as compared to WT A549 cells. To confirm these results, we performed single-cycle high MOI kinetics assays and observed reduced H1N1 virion production over time from (NOG)^−^ TKO cells as compared to WT A549 cells (Fig. 5D). Next, we performed multi-cycle replication assays and observed a 2–3 log decrease in viral titers over time for H1N1 in (NOG)^−^ TKO cells as compared to WT A549 cells (Fig. 5E). In contrast, H5N1 virus showed robust replication in (NOG)^−^ TKO cells, with only a modest decrease in viral titers in (NOG)^−^ TKO cells as compared to WT A549 cells. Together, these data indicate that truncation of three glycan types decreased the susceptibility of (NOG)^−^ TKO cells to H1N1 infection, yet did not dramatically affect H5N1 infection.

### Various avian IAV strains show robust replication in [NOG]^−^ TKO A549 cells

**Fig 6:**
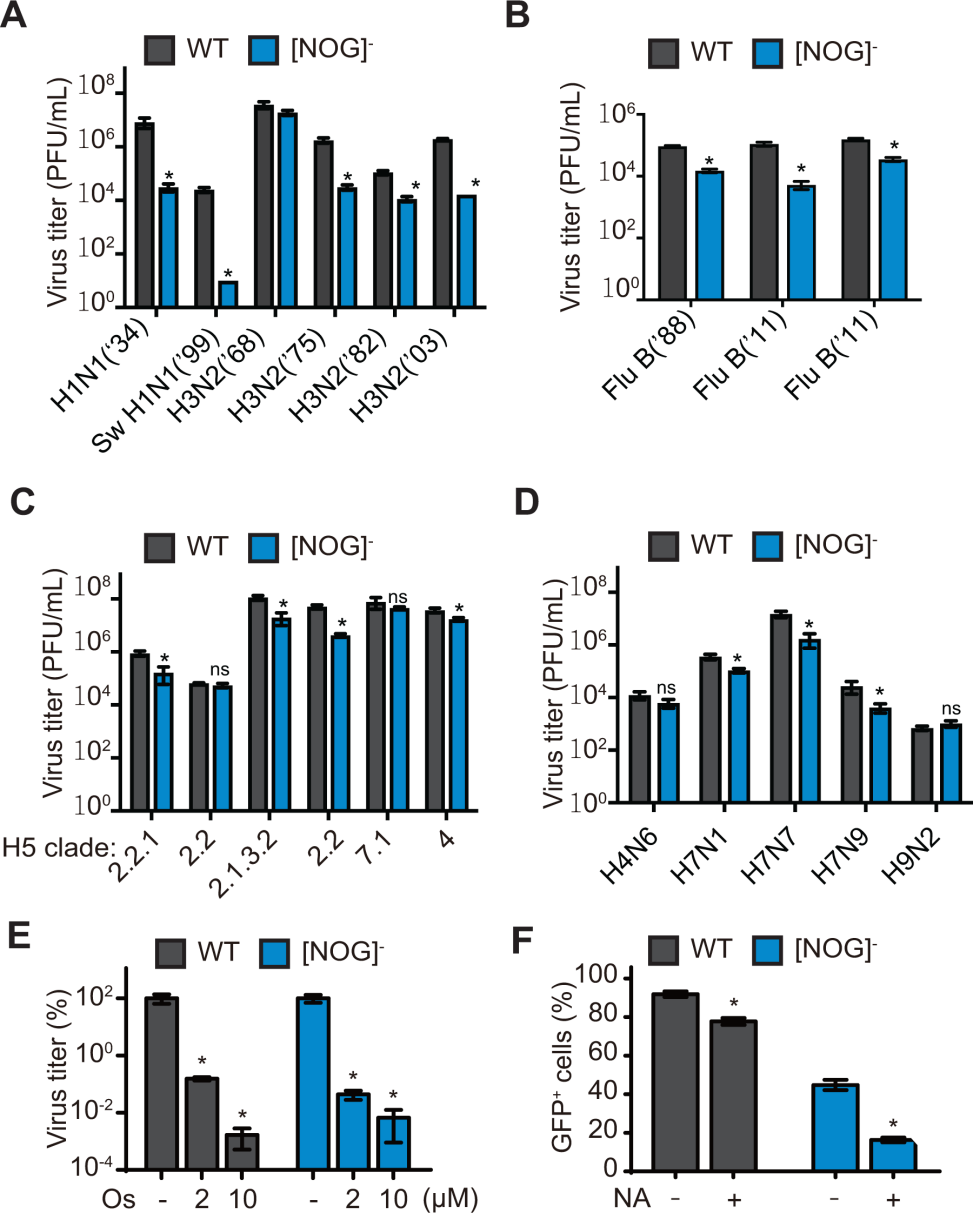
Avian but not human IAV strains show robust replication in [NOG]^-^ TKO A549 cells in a Sia dependent manner. (**A–D**) Multi-cycle replication assays with various IAV and influenza B strains in [NOG]^-^ TKO and WT A549 cells. (**A**) Replication of human and swine IAV strains. (**B**) Replication of human influenza B virus strains. (**C**) Replication of avian H5N1 viruses from different representative clades. (**D**) Replication of other avian IAV subytpes. Data are represented as mean titer of triplicate samples ± SD (PFU/mL). (**E**) Treatment with Oseltamivir carboxylate limits H5N1 replication in [NOG]^-^ TKO A549 cells. WT and TKO A549 cells seeded in 6-well dishes were infected at a low MOI with H5N1 (MOI = 0.001) and incubated with the indicated concentrations of Oseltamivir carboxylate (Os.) in the presence of TPCK-treated trypsin. At 48 hpi, viral titers in the supernatants were determined by plaque assay in MDCK cells. Data are represented as a percentage mean titer of triplicate samples relative to untreated cells ± SD. (**F**) Sialidase pretreatment decreases H5N1 infection in [NOG]^-^ TKO A549 cells. WT or [NOG]^-^ TKO A549 cells seeded in 6-well plates were pretreated with 500 mU/mL sialidase from *Clostridium perfringens* for 3 hours at 37°C before infection with H5N1-GFP (MOI = 3). At 16 hpi, the % of GFP positive cells was determined by flow cytometric analysis. Data are represented as mean percentage of GFP+ cells from triplicate samples ± SD. *Denotes *P*-value ≤ 0.05. ns is non-significant. Data are representative of at least two independent experiments.

Next, we expanded the panel of influenza viruses and tested the replication of a variety of influenza A and B viruses in (NOG)^−^ TKO A549 cells (Fig. 6A through D). We observed a 1–3 log decrease in the replication of human and swine H1N1 and H3N2 subtypes as well as influenza B viruses, with the exception of the 1968 pandemic H3N2 virus that originated from an avian host (Fig. 6A and B). The 1968 H3N2 strain showed robust replication in (NOG)^−^ TKO cells at levels comparable to WT A549 cells (Fig. S6). Interestingly, avian IAV replication of H4, H5, H7, and H9 subtypes remained high in (NOG)^−^ TKO cells (Fig. 6C and D; Fig. S6). Several avian strains did not show significant differences in replication between WT A549 and (NOG)^−^ TKO cells (H5 clades 1, 2.2, 7.1, H4N6, and H9N2). Taken together, our findings show that human and swine IAV strains require Sia on one of the three major glycoconjugates for viral entry, whereas avian IAV strains efficiently utilize an expanded repertoire of shorter sialoglycans.

### H5N1 IAV replication in [NOG]^−^ TKO A549 cells is dependent on residual Sia

Our glycomic profiling of (NOG)^−^ TKO cells showed the presence of sialyl Tn and sialyl T antigens, which are not affected by the loss of C1GALT1. To demonstrate that avian H5N1 replication occurs in a Sia-dependent manner in (NOG)^−^ TKO cells, we performed multi-cycle replication assays in the presence of viral neuraminidase inhibitor (Oseltamivir carboxylate). In this way, we can determine if IAV virions produced from (NOG)^−^ TKO cells require neuraminidase for release. We observed a >4 log decrease in H5N1 titers upon treatment with Oseltamivir for both (NOG)^−^ TKO and WT A549 cells, indicating that viral neuraminidase activity is essential for H5N1 replication in (NOG)^−^ TKO cells (Fig. 6E). In addition, pretreatment of (NOG)^−^ TKO cells with *C. perfringens* sialidase for 2 hours dramatically reduced single-cycle H5N1 infection in (NOG)^−^ TKO cells, validating the Sia-dependent replication of H5N1 in these TKO cells (Fig. 6F). Together, our studies demonstrate that avian H5N1 can efficiently utilize Sia moieties on minimally expressed shorter glycoconjugates.

### Validation of IAV glycoconjugate requirements in primary human airway CRISPR KO cells

**Fig 7:**
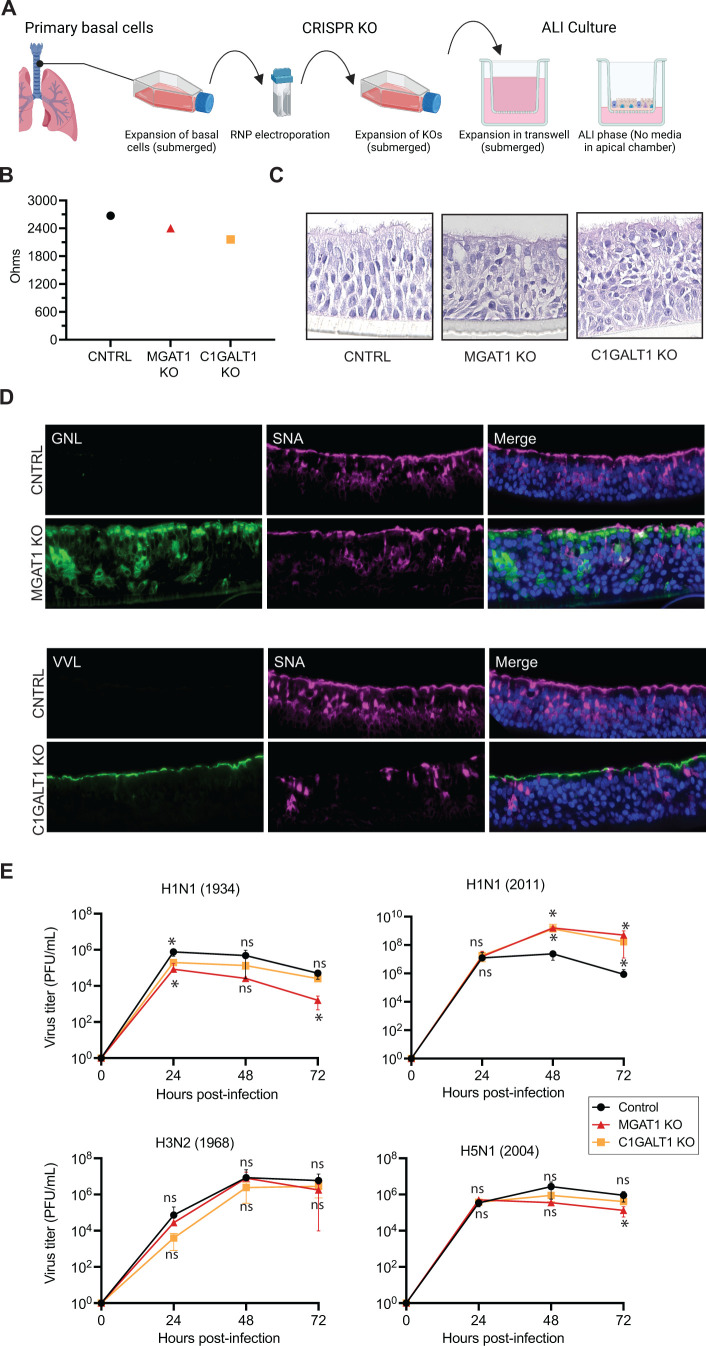
Replication of human and avian IAV strains in primary human airway cells with truncated N- or O- glycans. (**A**) Schematics for establishment of ALI cultures with polyclonal CRISPR KO basal cells. Primary human airway basal cells were electroporated with CRISPR RNP complex and allowed to expand. After ~2 to 3 days of expansion, approximately 2 × 10^5^ polyclonal KO cells were seeded on 24-well sized transwells and allowed to expand for 3 days in expansion media. On day 3, cells were switched to ALI culture conditions by removing media from the apical chamber and maintained in ALI conditions for at least 2 weeks prior to performing experiments. (**B**) TEER values in ALI cultures at 2 weeks. Values are presented in Ohms. (**C and D**) Two-week-old ALI cultures were fixed in paraformaldehyde, embedded in paraffin, and sliced into thin sections. (**C**) Histological analysis of ALI cultures by hematoxylin and eosin (H&E) staining. (**D**) Lectin staining of ALI sections. (**E**) Replication of various IAV strains in indicated primary KO cells. ALI cultures were infected with the indicated IAV strains and apical washes were collected at the indicated time points. Viral titers were measured by plaque assay in MDCK cells. Data is a pooled average of two independent experiments performed with two biological replicates. *Denotes *P*-value ≤ 0.05. ns is non-significant.

Prior glycomic profiling of human lung tissues showed the presence of taller polyLN containing N-glycan structures with extensive branching, which was not observed in WT A549 cells (15, 17, 20, 21). To investigate the role of different sialyl glycoconjugate types in a primary airway cell model, we established air-liquid interface (ALI) cultures using primary airway basal cells from the large conducting airways of normal donor lungs that were unsuitable for transplantation (Fig. 7A). Well-differentiated ALI cultures are a widely accepted primary airway epithelial cell model that mimics surface epithelium of the large conducting airways in the respiratory tract, as they form tight junctions and contain all the cell types including basal progenitor cells, multiciliated cells, secretory and goblet cells, and other rare cell types (28). To generate N- or O-glycan KO primary airway cells, primary basal cells were electroporated with CRISPR ribonucleoprotein complex containing sgRNA and recombinant Cas9 protein as demonstrated (29). After ~2 to 3 days of expansion, we assessed the efficiency of glycan KO by flow cytometry based lectin binding assays, and observed ~50% and ~85% KO efficiencies for *MGAT1* and *C1GALT1* sgRNAs, respectively (Fig. S7). At the same time, polyclonal CRISPR KO basal cells were seeded and differentiated into ALI cultures on a 24-well Transwell system. At 2 weeks post ALI differentiation, both *MGAT1* and *C1GALT1* polyclonal KO cells displayed properties similar to Control (CNTRL) cells, forming a well-differentiated multi-layer cell population with cilia (~4 to 6 layers) and exhibited transepithelial electrical resistance (TEER) values of ~1,800 to 2,200 Ω (versus 200 Ω blank) as anticipated (Fig. 7B and C). However, hematoxylin and eosin (H&E) staining showed some morphological differences between CNTRL and KO cells, an observation that will be investigated in the future. GNL and VVL lectin staining demonstrated that the majority of the differentiated cells lacked the intended glycans (Fig. 7D). Interestingly, unlike A549 (O)^−^ KO cells, ALI differentiated *C1GALT1* KO cells showed reduced SNA staining, which may be due to the complex nature of various cell types in these cultures. Next, we compared the replication of various human and avian IAV viruses in ALI cultures, and observed robust replication of both human and avian IAV strains in primary KO cells at levels similar to CNTRL cells (Fig. 7E). It should be noted that the viral titers for the H1N1 (1934) strain were approximately five-fold lower in *MGAT1* KO cells, indicating strain specific differences in glycoconjugate preference in the ALI culture system. Taken together, these results in primary human airway KO cells further validate our findings that IAV strains can utilize any one of the major glycoconjugate types for host cell infection.

## DISCUSSION

In this study, we assessed the importance of various cell surface sialylglycoconjugate types to human versus avian IAV infection. Using the CRISPR/Cas9 gene editing technique, we truncated the three major types of glycoconjugates (N-glycans, O-glycans, and GSL), either individually or concurrently, in a human lung epithelial cell line (A549) and evaluated IAV replication. Our studies demonstrated that both human and avian IAV strains did not show strict preferences for any of the three types of glycoconjugates for infection in A549 cells. Interestingly, our studies in (NOG)^−^ TKO A549 cells showed that concurrent truncation of the three major glycoconjugates significantly reduced human H1N1 and H3N2 replication, indicating that human IAV strains require the presence of Sia on one of the three major glycoconjugates. In contrast, several avian IAV strains (H4, H5, H7, and H9 subtypes) demonstrated robust replication in (NOG)^−^ TKO A549 cells. Glycomic profiling of (NOG)^−^ TKO A549 cells showed the presence of other less prevalent shorter sialoglycans, indicating that avian IAV strains can also utilize these shorter glycoconjugates for infection. Taken together, our studies reveal that, unlike human IAV strains, avian IAV strains demonstrate remarkable plasticity in glycoconjugate receptor usage in human lung cells.

It has been well established that avian and human IAV strains differ in Siaα2–3Gal versus Siaα2–6Gal receptor preferences (8, 9). However, it remains unknown if a specific type of glycoconjugate serves as the primary receptor or if multiple glycoconjugate types are capable of facilitating IAV entry. Much of our understanding of glycoconjugate preferences for various IAV strains has been inferred from *in vitro* binding studies using virions or purified HA protein on glycan array slides (11, 13 – 17) (19 – 21). Studies with CFG arrays and shotgun lung tissue glycan arrays indicated that human adapted IAV strains preferred longer branched polyLN glycans (N-glycans) for attachment. Interestingly, some of the parental pandemic IAV strains originating from avian hosts bound to short sialyl-LN glycans, suggesting that avian IAV strains may adapt to utilize polyLN in humans. Our studies in CRISPR KO A549 cells demonstrate that both human and avian IAV can utilize all three major types of glycoconjugates for host cell infection. Surprisingly, in (NOG)^−^ TKO A549 cells lacking all three major glycoconjugate types, we observed robust replication of several subtypes of avian IAV strains, albeit with a modest reduction in replication of certain IAV strains (Fig. 6). However, several human and swine IAV strains showed significantly decreased replication (~1 to 3 log lower) in these (NOG)^−^ TKO A549 cells, with the exception of the 1968 pandemic H3N2 virus, which originated from an avian host (Fig. 6). Of note, the H1N1 (1934) PR8 strain, a mouse adapted human IAV strain, has been shown to bind both Siaα2–3Gal and Siaα2–6Gal receptors; despite Siaα2–3Gal receptor binding, the replication phenotype of the H1N1 (1934) strain closely resembled that of other human IAV strains in CRISPR KO cells. Comparative glycomic profiling of (NOG)^−^ TKO and WT A549 cells showed complete loss of N-glycans as well as Core 1 and 2 O-glycans, as intended by our CRISPR KO approach. As anticipated, we detected other less prevalent O-glycan Cores such as sialyl Tn antigen and sialyl T antigens, which are shorter sialoglycans lacking N-acetyllactosamine (LN) units that are unaffected by the loss of C1GALT1. These findings demonstrate that avian IAV strains can efficiently access and utilize less predominant shorter sialoglycans for host cell infection. Alternatively, as truncation of the three major glycoconjugate types in (NOG)^−^ TKO A549 cells reduced the cell surface availability of sialoglycans (~0.3% N-glycans and 7% O-glycans in TKO cells), it is possible that human IAV strains require a higher Sia receptor density for host cell binding and infection as compared to avian IAV strains. This interpretation is in agreement with our previous study in which avian H5N1 virus showed robust replication in A549 cells cultured in the presence of 3Fax-Neu5Ac, a competitive inhibitor of sialyltransferases that decreases cell surface Sia density (30). In contrast, H1N1 and H3N2 viruses showed a >3 log reduction in viral titers in 3Fax-Neu5Ac treated A549 cells. Thus, our studies demonstrate that the receptor requirements of avian versus human IAV strains extend beyond the well-established Siaα23 versus Siaα2,6 linkage preferences.

Prior studies demonstrate that the glycome of human lungs is composed of taller and extensively branched polyLN repeat containing N-glycans and LN repeat containing O-glycans, which are distinct from the glycome of lung cell lines (e.g., A549 cells) (19 – 21). Our glycomic profiling of WT A549 cells showed the presence of short branched N-glycans with one or two LN repeats and single LN containing O-glycans (Fig. 4); however, taller branched polyLN were not detected in A549 cells, confirming the differences in the glycome of primary cells versus transformed A549 cell line. In the primary human lung ALI cultures, we did observe reduced replication of the H1N1(1934) strain in *MGAT1* KO cells, which will be investigated in the future. As ALI cultures are composed of multiple cell types, it will be interesting to see if there are any changes in IAV tropism in KO ALI cultures. In addition, we plan to assess IAV replication in DKO and TKO ALI cultures. Together, these studies demonstrate that avian IAV strains can utilize a broader repertoire of glycoconjugate receptors as compared to human IAV strains for host cell infection. This remarkable plasticity in sialoglycan receptor usage may allow avian IAV strains to readily infect other host species.

In summary, our studies in CRISPR glycoengineered human lung epithelial cells highlight the stark differences in glycoconjugate receptor preferences for avian versus human and swine IAV strains. Human IAV strains required one of the three major types of glycoconjugates for efficient host cell infection, with some preference for N-glycans. In contrast, avian IAV strains demonstrated versatility in glycoconjugate receptor usage, as these strains replicated efficiently in cells expressing shorter sialoglycans such as sialyl Tn and Sialyl T antigens. Taken together, our data indicate that human IAV strains may rely on a limited repertoire of sialylglycoconjugates for host cell infection, whereas avian IAV strains can utilize diverse sialylyglycoconjugates for viral entry. These findings will have important implications for our understanding of how glycoconjugate receptor usage determines the host range of zoonotic IAV strains.

## MATERIALS AND METHODS

### Cells and viruses

Human lung epithelial (A549) cells and African green monkey kidney (Vero) cells were cultured in DMEM (Gibco) supplemented with 10% fetal bovine serum (FBS, Atlanta Biologicals) and 1% penicillin/streptomycin (P/S). Nuli-1 cells were cultured on Collagen IV coated plates in Bronchial Epithelial Cell Growth Media (BEGM) with supplements (Lonza) as previously described (27). Madin-Darby canine kidney (MDCK) cells were cultured in MEM supplemented with 10% FBS and 1% P/S.

IAV strains used in this study were obtained from different sources—A/Puerto Rico/8/1934 (H1N1, Mount Sinai), high virulent PR8 (hvH1N1 provided by Dr. Georg Kochs), A/Hong Kong/1/1968 (HK68, H3N2), A/Wyoming/03/2003 (H3N2), A/Victoria/3/1975 (H3N2), A/Philippines/2/1982 (H3N2), A/Swine/Minnesota/37866/1999 (SwH1N1), low pathogenic version of A/Vietnam/1203/2004 (H5N1-low pathogenic), low pathogenic version of A/Netherlands/213/2003 (H7N7-low pathogenic, kindly provided by Dr. Ron Fouchier), A/blue-winged teal/Illinois/10OS1563/2010 (H4N6), A/Rhea/North Carolina/39482/1993 (H7N1), A/shorebird/Delaware Bay/127/2003 (H9N2), and A/Anhui/1/2013H7N9 2:6 PR8 reassortant virus [H7N9 (PR8)]. H1N1-GFP (PR8) and H5N1-GFP (low pathogenic) viruses were grown as previously described (31, 32). Influenza B strains used in this study—B/Yamagata/16/1988, B/Nevada/03/2011, B/Texas/06/2011. Several of the other influenza virus strains were obtained through BEI Resources or generated by reverse genetics system (33). All influenza viruses were either grown in embryonated eggs or MDCK cells. Influenza viruses were aliquoted and stored at −80°C before titering by plaque assay on MDCK cells using 2.4% Avicel RC-581 (a kind gift from FMC BioPolymer, Philadelphia, PA). Viral plaques were quantified 2–3 days post-infection by crystal violet staining. Vesicular stomatitis virus expressing GFP, kindly provided by Dr. Glenn Barber at the University of Miami, FL, was propagated in Vero cells, and titers were determined by plaque assay on Vero cells using 1% methylcellulose (Sigma) (34).

### Generation of CRISPR KO cells

*MGAT1* and *C1GALT1* KO A549 cells were generated using the lentiCRISPR v2 (#52961, Addgene) single vector system with Puromycin selection as previously described (35, 36). *MGAT1* single KO cells were used to generate *MGAT1/C1GALT1* DKO cells and *MGAT1/UGCG* DKO cells with the pLentiSpBsmBI sgRNA Hygro vector (#62205, Addgene). *MGAT1/C1GALT1* DKO cells were used to generate *MGAT1/C1GALT1/UGCG* TKO cells with the lentCRISPRv2 neo vector (#98292, Addgene). Primers for sgRNA target sites used here are listed in Table S1. On day 2 post-lentivirus transduction, A549 cells were subjected to drug selections for ~14 days at the following concentrations: puromycin - 2 µg/mL (Invivogen), hygromycin - 800 µg/mL (Invitrogen), neomycin (G418) - 800 µg/mL (Invitrogen). Clonal knockout cells were isolated by seeding ~100 cells in a 150 mm plate and allowing them to grow as individual colonies. Successful KO clones were initially identified by flow cytometry using lectins, and subsequently confirmed by Western blot analysis for loss of MGAT1 and/or C1GALT1 expression. In addition, disruption of the intended target sites was confirmed by Sanger sequencing of the region flanking the sgRNA target as previously described (Tables S1 and S2) (30). Identified insertion and deletion mutations at the sgRNA target sites are listed in Table S2.

### Virus infections

For assessment of single cycle virus infections, cells were seeded at a density of 3 × 10^5^ cells per well in a 12-well plate, and infection with GFP viruses was performed in infection media [DMEM supplemented with 0.2% bovine serum albumin (BSA) (MP Biomedicals) and Penicillin/Streptomycin (Corning)] without the addition of TPCK-treated trypsin. At 16 hours post-infection, cells were trypsinized and prepared for flow cytometric analysis. Data were acquired on a BD FACSVerse instrument and analyzed using FlowJo software. For assessment of multi-cycle virus replication, WT and knockout A549 cells were seeded in triplicate at a density of 8 × 10^5^ cells per well in a 6-well plate. On the next day, cell numbers were measured prior to infection. Cells were washed twice with phosphate buffered saline (PBS) and inoculated with virus at the indicated MOI in infection media supplemented with 0.9 µg/mL TPCK-treated trypsin (T1426, Sigma). The inoculum was removed after incubation for 1 hour at 37°C, and cells were washed twice with PBS before addition of fresh infection media. Supernatants were collected at the indicated time points and stored at −80°C, and viral titers were measured by plaque assay. VSV infections were performed as above with infection media without TPCK-treated trypsin, and supernatant titers were assessed by plaque assay on Vero cells using 1% methylcellulose (Sigma) (DMEM, 2% FBS, P/S).

### Sialidase and oseltamivir carboxylate treatment

For sialidase pretreatment experiments, cells seeded in 12-well plates were pretreated with 500 mU/mL α2-3/6/8 sialidase from *Clostridium perfringens* (Roche, 11585886001) for 3 hours at 37°C before infection. For viral neuraminidase inhibitor treatment experiments, Oseltamivir carboxylate (kind gift from Roche) was added at the indicated concentrations to the infection media after 1 hour post-virus infection.

### Western blot analysis

Whole cell extracts were prepared using RIPA buffer containing protease inhibitors (Roche, 1187358001), and Western blot analysis was performed with ~80 µg of total protein as previously described (30). Anti-MGAT1 (ab180578 Rabbit monoclonal) and anti-C1GALT1 (ab237734 Rabbit polyclonal) antibodies for Western blot analysis were purchased from Abcam and used at a 1:1,000 dilution.

### Lectin and antibody staining

Fluorescein labeled *Galanthus Nivalis* Lectin (GNL-FITC, #FL-1241 1:250), Cy3 labeled *Sambucus Nigra* Lectin (SNA-Cy3, #CL-1303, 1:500), and fluorescein labeled *Vicia Villosa* Lectin (VVL-FITC, #FL-1231, 1:500) were purchased from Vector Laboratories. FITC-conjugated Cholera Toxin B subunit (CTB-FITC, #C1655, 1:250) was purchased from Sigma-Aldrich. Anti-Sialyl Tn antibody was purchased from ThermoFisher. Cells were incubated with the fluorescently labeled lectins for 30 minutes on ice in lectin staining buffer (PBS supplemented with 0.2% BSA and 0.1 mM CaCl_2_) and excess unbound lectin was removed by washing in the same buffer. Data were acquired on a BD FACSVerse flow cytometer and analysis was performed using FlowJo Software.

### HA binding assay

Cell surface binding of HA proteins was performed with purified recombinant H1 HA and H5 HA (BEI Resources). Briefly, 1 × 10^6^ cells were incubated with 5 µg of HA on ice for 1 hour, and the unbound protein was removed by washes with staining buffer (PBS supplemented with 0.2% BSA and 2 mM EDTA). Cells were then fixed with 4% paraformaldehyde for 10 minutes at RT and washed with PBS. Non-specific secondary antibody binding to cells was blocked using blocking buffer (staining buffer supplemented with 5% normal goat serum and 0.1% Tween 20) for 15 minutes. The amount of bound HA was measured using anti-H1N1 rabbit sera or anti-H5N1 mouse sera for 1 hour, followed by a secondary goat antibody conjugated with Alexa Fluor 647 (Invitrogen) for 30 minutes. All blocking and staining steps were performed on ice. Cells were analyzed by flow cytometry on a BD FACSVerse flow cytometer; data analysis was performed using FlowJo Software. Relative binding efficiency was calculated as follows: Relative binding (%) = [(MFI binding/MFI background)/(MFI WT cell binding/MFI WT cell background)] × 100.

### Cell surface virion binding assay

Measurements of cell surface binding by IAV particles were performed with 1 × 10^6^ cells at an MOI = 100. Binding of virions was carried out on ice for 1 hour 30 minutes, and the unbound virions were removed by extensive washes with staining buffer (PBS supplemented with 0.2% BSA and 2 mM EDTA). Cells were then fixed with 4% paraformaldehyde for 10 minutes at room temperature and washed with PBS. The levels of virion binding were measured by cell surface staining with anti-H1N1 rabbit sera or anti-H5N1 mouse sera. Briefly, cells were blocked with a staining buffer for 15 minutes, incubated with anti-H1N1 rabbit sera or anti-H5N1 mouse sera (1:500) for 1 hour, washed with blocking buffer to remove unbound antibodies, then subsequently incubated with a secondary goat antibody conjugated with Alexa Fluor 488 (Invitrogen) for 30 minutes. Data acquisition was performed on a BD FACSVerse flow cytometer and analysis was performed using the FlowJo software. Relative binding efficiency was calculated as follows: Relative binding (%) = [(MFI binding/MFI background)/(MFI WT cell binding/MFI WT cell background)] × 100.

### Glycomics profiling

Analyses for N- and O-glycans were performed at the Complex Carbohydrate Research Core at the University of Georgia–Athens, using previously reported methods (37, 38). For analysis of glycan structures, WT and (NOG)^−^ TKO A549 cells were homogenized 55°C for 30 minutes. Excess salts were removed with a prewashed 10 kDa MWCO filter and were exchanged into 50 mM ammonium bicarbonate. The protein fraction at the top of the MWCO filter were removed and resuspended into 50mM ammonium bicarbonate before treatment with PNGase F (NEB, P0709) for 20 hours at 37°C. The sample was passed through another 10 kDa MWCO filter separating the released N-glycans into the flow through. The remaining proteins containing O-linked glycans were removed from the top of the filter before treatment with 1 M sodium borohydride in 50 mM sodium hydroxide. O-glycans were released via beta-elimination through this procedure at 45°C overnight before neutralization with 10% acetic acid, desalting with Dowex (50X×8−100, Sigma Aldrich, St. Louis, MO) then lyophilized to dryness. Borates were removed with repeated additions of 9:1 methanol:acetic acid under a nitrogen flow before resolubilization of the material and passage through a C18 SPE cartridge to further purify the material before permethylation and analysis.

Permethylation and MS Analysis: N- and O-linked glycans were permethylated using previously described methods (37). Briefly, dried samples were reconstituted in DMSO and treated with a mixture of methyl iodide in a DMSO/NaOH slurry. After quenching the reaction with the addition of water, the permethylated analytes were extracted out of the solution by addition of dichloromethane. Mass spectrometry analysis was carried out by LC-MS. Permethylated glycans were reconstituted in a 50:50 MeOH:H2O mixture containing 1 mM NaOH which acts as a charge carrier. The solution was analyzed on a Thermo Orbitrap Fusion Tribrid Mass Spectrometer with direct infusion at a flow rate of 1–2 µL/minute and analyzed in positive ion mode. An automated method that collects a full MS spectra at 120,000 resolution followed by data dependent MS/MS fragmentations (CID) at 60,000 resolution was used to collect data for the glycosphingolipids. Data for the permethylated N- and O-glycans were collected by LC-MS on a Thermo Orbitrap Fusion Tribrid Mass Spectrometer in line with an Ultimate 3000RSLCnano LC-system. A commercial C18 column of 15 cm × 75 µm id filled with 3 µm C18 material was used for analysis (Thermo Fisher; Cat 164568). Injections of permethylated N- or O-linked glycans were carried out from low to high acetonitrile in water with 1 mM sodium acetate. The precursor ion scan was acquired at 120,000 resolution in the Orbitrap analyzer and precursors at 3 seconds were selected for MS/MS fragmentation (CID fragmentation) in the Orbitrap at 30,000 resolution analyzer and precursors at 3 seconds were selected for MS/MS fragmentation (CID fragmentation) in the Orbitrap at 30,000 resolution.

### Generation of primary airway CRISPR KO cells

Primary human tracheal/bronchial airway epithelial cells (passage 0, P0), provided by the University of Iowa’s *In Vitro* Models and Cell Culture Core, were expanded on placental collagen-coated tissue culture 10 cm dishes in PneumaCult-Ex Plus Basal Media (STEMCELL Technologies). Cells were trypsinized using TrypLE (Gibco), washed with Dulbecco’s PBS without calcium/magnesium (Gibco), and centrifuged at 1,000 rpm for 5 minutes at RT. For each RNP nucleofection, RNPs were prepared first by combining the Cas9-V3 nuclease (IDT) with sgRNA (with tracer RNA) (IDT) at a 1:5 molar ratio and allowed to form a complex for 20 minutes at room temperature. After incubation, 2 µL of RNP mixture was added to 5 × 10^5^ cells resuspended in 98 µL nucleofection solution provided with the Amaxa Basic Nucleofector Kit for Primary Mammalian Epithelial Cells (Lonza). This primary cell-RNP mixture was transferred into cuvettes, and electroporated using the Lonza Nucleofector 2b (Lonza) with the U-024 program. After nucleofection, cells were transferred into 6-well collagen-coated dishes with prewarmed PneumaCult-Ex Plus Basal Media and incubated at 37°C. At near-confluency (P1), these cells were expanded in 10 cm tissue culture dishes (P2) and subsequently, 2 × 10^5^ cells were seeded onto collagen-coated polycarbonate transwell filters (size 6.5 mm, 0.4 um pore size, Corning). At day 3 post-seeding, cells were allowed to differentiate at an air-liquid interface (ALI) by placing the inserts in PneumaCult ALI differentiation media and removing the media from the apical chamber. Control non-manipulated primary cell cultures (P2) were seeded and differentiated similarly. To assess KO efficiency, an aliquot of RNP electroporated basal cells was stained with fluorescein (FITC)-conjugated lectins VVL and GNL (Vector Laboratories) diluted 1:500 in lectin staining buffer for 30 minutes on ice. Dead cells were excluded with a LIVE/DEAD Fixable Violet Cell Stain (Invitrogen). Cells were analyzed on the Attune NxT Flow Cytometer (Life Technologies), and the percent of editing efficiency was expressed as % VVL-FITC or GNL-FITC positive cells.

### TEER measurements of ALI cultures

Trans-epithelial electrical resistance (TEER) of differentiated airway epithelial ALI cultures were measured at 2 weeks post-seeding using the Epithelial Voltohmmeter (World Precision Instruments). Briefly, PneumaCult-ALI media was added to both the basolateral (600 µL) and apical chambers (200 µL), and the TEER was measured in Ohms using the STX2/“chopstick” electrodes. The TEER value for *trans* well inserts without cells was ~200 Ohms.

### Histological analysis of ALI cultures

Two-week-old ALI cultures were washed with DPBS and fixed by adding freshly prepared 4% paraformaldehyde to both the apical and basolateral chambers (200 µL and 400 µL, respectively). After extensive washing with DPBS, the transwell membrane was cut out and embedded in a paraffin block. Thin sections of paraffin embedded cells placed on a glass slide were subjected to H&E and lectin staining. Images were taken at 40× magnification on a Keyence BZ-X810 All-in-One Fluorescent Microscope using the Keyence BZ Series software.

### Statistical analysis

Statistical significance was determined by Student’s *t*-test, and *P*-values ≤ 0.05 were considered significant and denoted with an asterisk. Non-significant values are denoted as ns.

## AFTER EPUB

*[This article was published on 16 October 2023 with Lauren Pepi's name misspelled in the byline. The spelling was corrected in the current version, posted on 1 November 2023.]*

## SUPPLEMENTAL MATERIAL

Supplemental information jvi.00906-23-s0001.pdf Fig. S1 to S7 and Tables S1 to S3.

ASM does not own the copyrights to Supplemental Material that may be linked to, or accessed through, an article. The authors have granted ASM a non-exclusive, world-wide license to publish the Supplemental Material files. Please contact the corresponding author directly for reuse.
