# Supplementary material for: Avian influenza A viruses exhibit plasticity in sialylglycoconjugate receptor usage in human lung cells
Source: J Virol. 2023 Oct 16;97(11):e00906-23. doi: 10.1128/jvi.00906-23 (PMC10688379; doi:10.1128/jvi.00906-23)
Supplement: Supplemental information — Fig. S1 to S7 and Tables S1 to S3. [file jvi.00906-23-s0001.pdf]

Avian Influenza A Viruses Exhibit Plasticity in Sialylglycoconjugate Receptor Usage in Human Lung Cells

Chieh-Yu Liang et al,

Supplementary figure legends

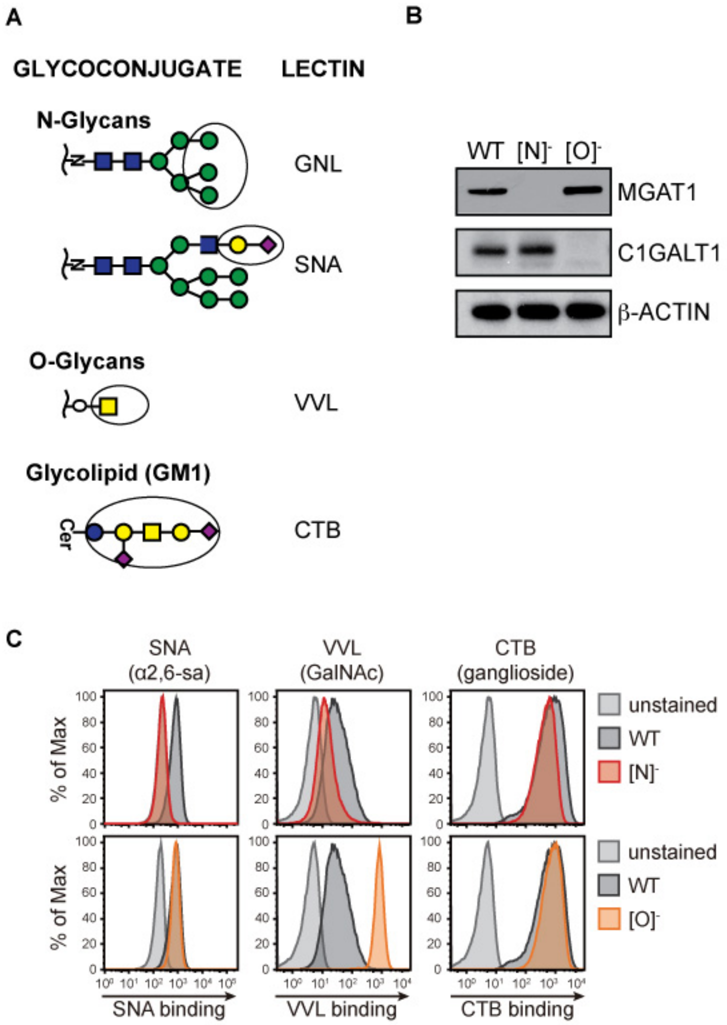

Figure S1. Characterization of $[N]^-$ and $[O]^-$ KO A549 cells. (A) Binding specificities of different lectins used in this study. GNL - *Galanthus Nivalis* Lectin, SNA -*Sambucus Nigra* Lectin, VVL - *Vicia Villosa* Lectin, and CTB - Cholera Toxin

B subunit. (B) Western blot analysis of MGAT1 and C1GALT1 expression in KO A549 cells. β-actin levels are shown as loading controls. (C) Comparison of the binding properties of various lectins in WT and KO A549 cells. Representative flow cytometry plots showing differences in lectin binding. Data are representative of at least two independent experiments.

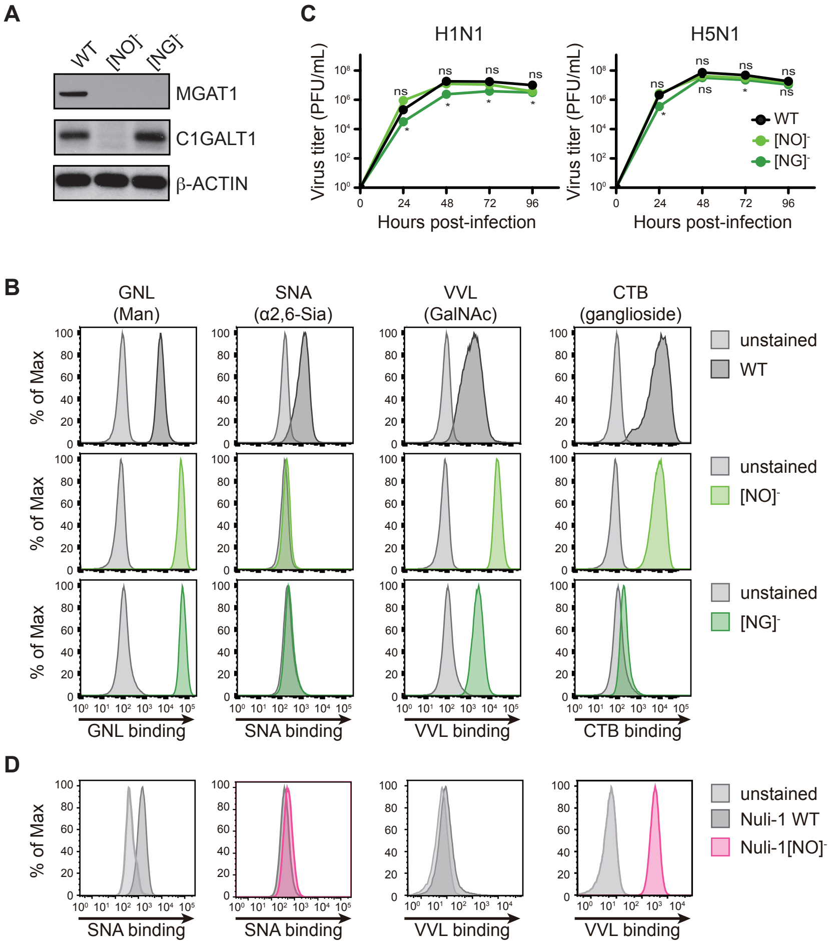

Figure S2. Characterization of DKO A549 and DKO Nuli-1 cells. (A) Western blot analysis of MGAT1 and C1GALT1 expression in DKO A549 cells. β-actin levels are shown as loading controls. (B) Comparison of the binding properties of various lectins in WT and DKO A549 cells. Representative flow cytometry plots showing differences in lectin binding. Data are representative of at least two

independent experiments. (C) Multi-cycle replication assays with H1N1 and H5N1 in [NO]$^-$ and [NG]$^-$ DKO A549 cells. WT and DKO A549 cells seeded in 6-well dishes were infected at a low MOI with H1N1 (MOI=0.01) or H5N1 (MOI=0.001) in the presence of TPCK-treated trypsin and viral titers in the supernatants at different hpi were determined by plaque assay in MDCK cells. Data are represented as mean titer of triplicate samples ± SD (PFU/mL). * denotes p-value ≤0.05. ns is non-significant. Data are representative of at least two independent experiments. (D) Comparison of the binding properties of various lectins in WT and [NO]$^-$ DKO Nuli-1 cells. Representative flow cytometry plots showing differences in lectin binding. Data are representative of at least two independent experiments.

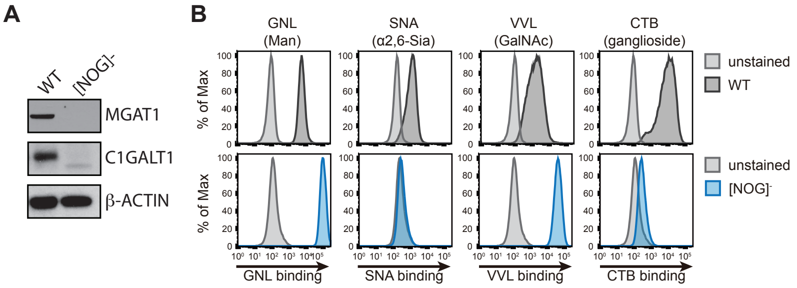

Figure S3. Characterization of [NOG]$^-$ TKO A549 cells. (A) Western blot analysis of MGAT1 and C1GALT1 expression in [NOG]$^-$ TKO A549 cells. β-actin levels are shown as loading controls. (B) Comparison of the binding properties of various lectins in WT and [NOG]$^-$ TKO A549 cells. Representative flow cytometry plots showing differences in lectin binding. Data are representative of at least two independent experiments.

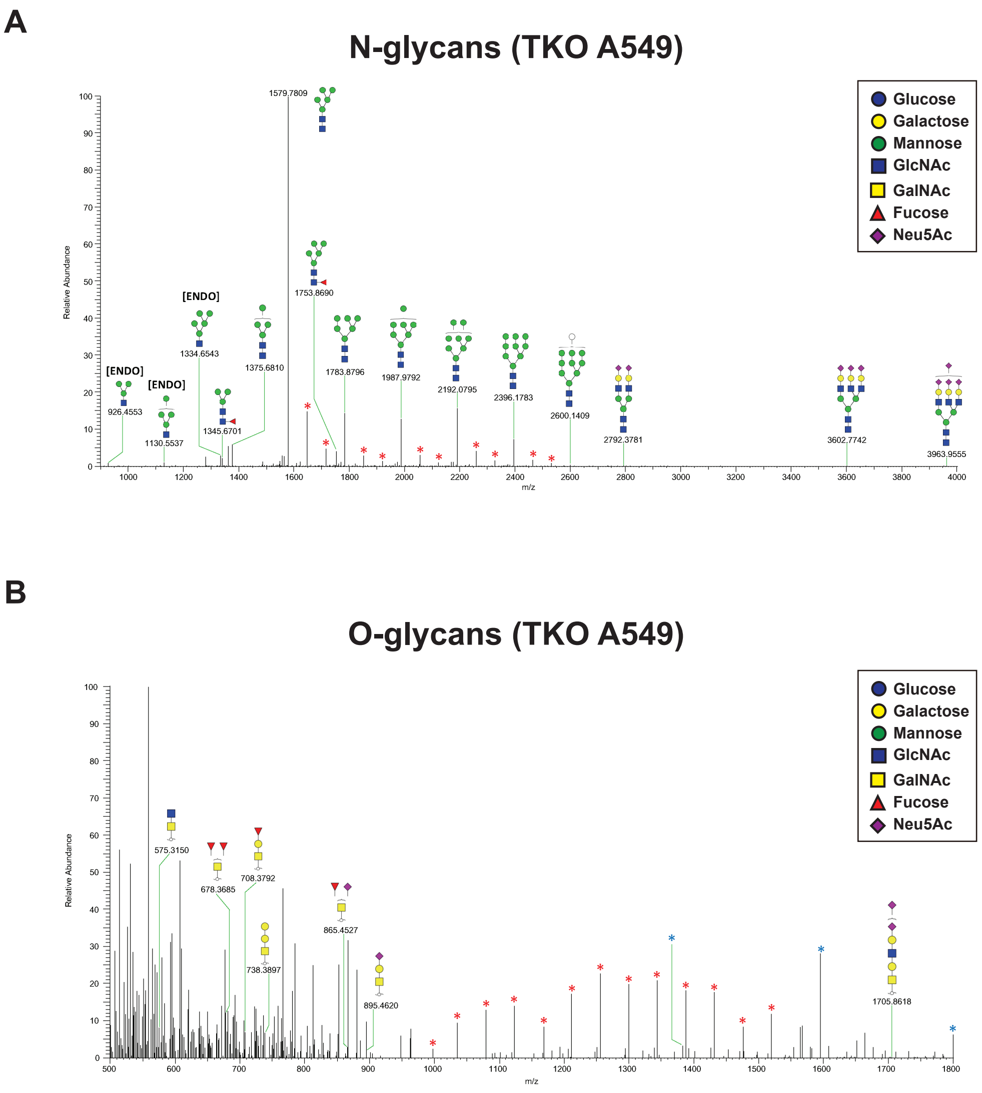

Figure S4. Glycomic profiling of [NOG]$^-$ TKO A549 cells. (A) ESI-MS spectrum of N-glycans expressed in [NOG]$^-$ TKO cells. Red asterisks indicate [M+$NaCHO_2$] or [M+2$NaCHO_2$] adduct of an already displayed glycoform. (B) ESI-MS spectrum of N-glycans expressed in [NOG]$^-$ TKO A549 cells. Red asterisks indicate PEG contamination peak. Blue asterisks indicate reduced N-glycan mass.

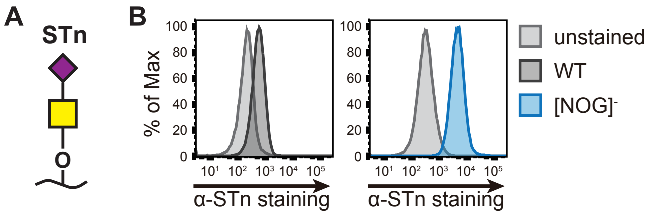

Figure S5. Assessment of Sia levels in [NOG]$^-$ TKO A549 cells. (A) Structure of sialyl Tn antigen. (B) Flow cytometry plots comparing STn antigen levels in WT and [NOG]$^-$ TKO A549 cells. STn levels were analyzed using an anti-STn antibody and representative histograms are shown.

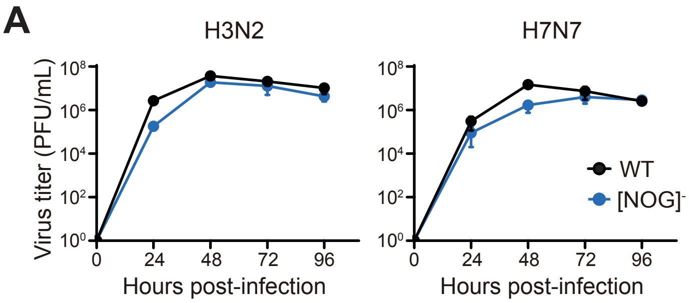

Figure S6. Assessment of H3N2 and H7N7 replication in [NOG]$^-$ TKO A549 cells. WT and TKO A549 cells seeded in 6-well dishes were infected at a low MOI with H3N2 or H7N7 (MOI=0.01) in the presence of TPCK-treated trypsin and viral titers in the supernatants at different hpi were determined by plaque assay in MDCK cells. Data are represented as mean titer of triplicate samples ± SD (PFU/mL). * denotes p-value ≤0.05. ns is non-significant. Data are representative of at least two independent experiments.

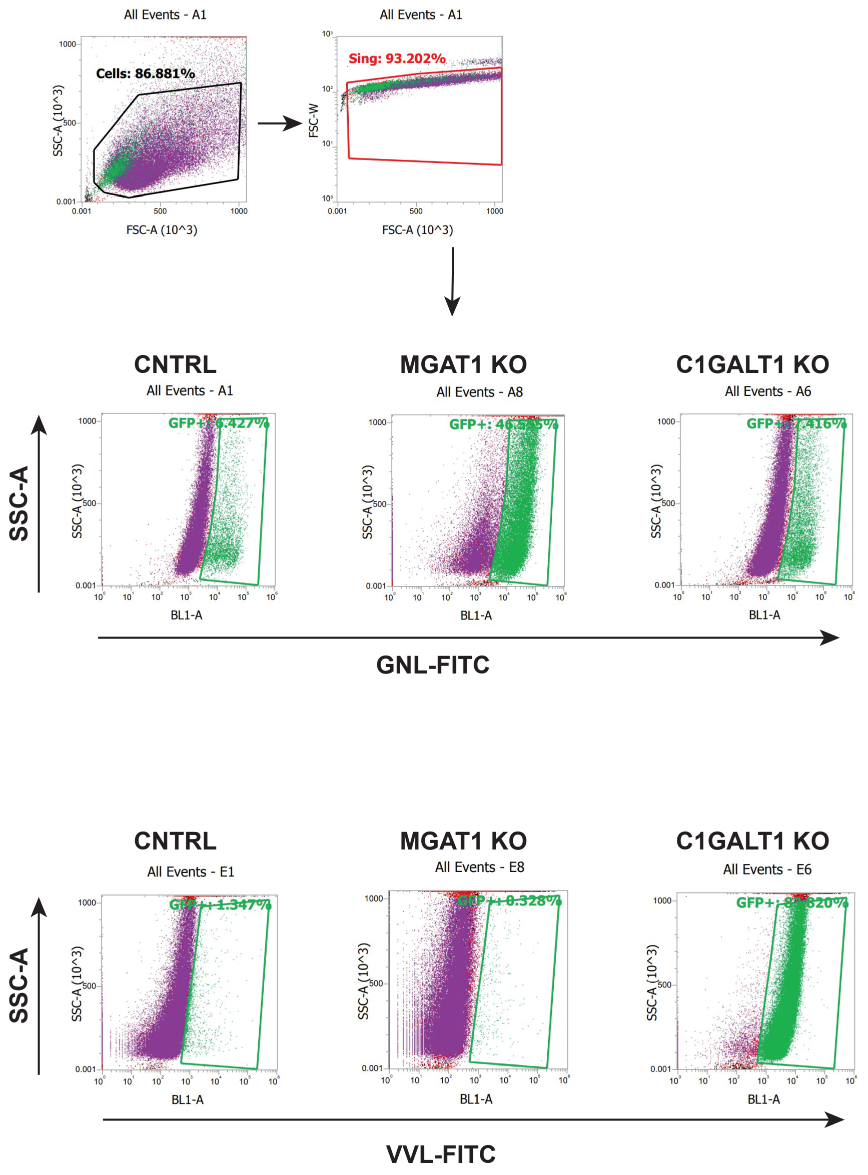

Figure S7. Lectin binding analysis of CRISPR KO primary airway cells. Primary airway basal cells were electroporated with CRISPR RNP and expanded. At passage #2, a portion of the cells were stained with FITC conjugated lectins (GNL-FITC, VVL-FITC) and analyzed by flow cytometry. Representative gating strategy used for flow cytometry is shown.

Table S1: Oligonucleotide sequences

Oligonucleotide Sequences for Generating Gene Specific sgRNA KOs.

Gene	Forward Primer	Reverse Primer
MGAT1	CACCGTGGGAATCACCGCCGGCGCG	AAACCGCGCCGGCGGTGATTCCCAC
C1GALT1	CACCGACAACACTTTGTTACAACGC	AAACGCGTTGTAACAAAGTGTTGTC
UGCG	CACCGTGGAGGGAATGGCCGTCTTC	AAACGAAGACGGCCATTCCCTCCAC

Primer Sequences for PCR Amplification of region surrounding the sgRNA Target Site.

Gene	Source	Forward Primer	Reverse Primer
MGAT1	gDNA	GCAGTCCTGGCTAACGATGA	CCTCACCCGGGAAGTGATTC
C1GALT1	gDNA	TATGACCGGCCCTCAAAACC	GCTCCTCCACTCATGTAGCC
UGCG	gDNA	TTTCCTCTCCCCACCTTCCT	AACGTTTCCCATTCTCGCCT

SgRNA Sequences for Generating Primary Airway Basal KOs.

Gene	sgRNA
MGAT1	GGGCATTCCAGGCCACAAAG
C1GALT1	ATCCTATTGCTGATCCACAG

Only protospacer region shown.

Table S2: Sequence Analysis of sgRNA Target Sites in Clonal CRISPR KO Cells.

Gene Name	Type of Mutation (In/Del)
**Single KO cells**	
MGAT1 KO	35nt Del/1nt In
C1GALT1 KO	106nt Del
**MGAT1/C1GALT1 DKO cells**	
MGAT1	35nt Del
C1GALT1	ND (confirmation by western blot /lectin binding assays)
**MGAT1/UGCG DKO cells**	
MGAT1	35nt Del
UGCG	1nt Ins/1nt Ins
**MGAT1/C1GALT1/UGCG TKO cells**	
MGAT1	35nt Del
C1GALT1	ND (confirmation by western blot /lectin binding assays)
UGCG	1nt Ins/16nt Del

Nucleotides = nt; Insertions = In; Deletions = Del; ND=not determined

Table S3: List of Virus Strains and Isolated Host Species

Virus strains	Isolated host species
H1N1, A/Puerto Rico/8/1934	Human (adapted in mice)
H3N2, A/Hong Kong/1/1968	Human
H3N2, A/Victoria/3/1975	Human
H3N2, A/Philippines/2/1982	Human
H3N2, A/Wyoming/03/2003	Human
H1N1, A/Swine/Minnesota/37866/1999	Swine
H4N6 A/blue-winged teal/Illinois/10OS1563/2010	Avian
H7N1,A/Rhea/North Carolina/39482/1993	Avian
H7N7, A/Netherlands/213/2003 (low pathogenic)	Avian (human isolate)
H7N9, A/Anhui/1/2013 (2:6 PR8 reassortant)	Avian (human isolate)
H9N2, A/shorebird/Delaware Bay/127/2003	Avian
H5N1, A/Vietnam/1203/2004 Clade1 (low pathogenic)	Avian (human isolate)
H5N1, A/Egypt/N03072/2010 Clade 2.2.1 (1:7 VN04) (low pathogenic)	Avian (human isolate)
H5N1, A/Indonesia/5/2005 Clade 2.1.3.2 (1:7 VN04) (low pathogenic)	Avian (human isolate)
H5N1, A/chicken/India/NIV33487/2006 Clade 2.2 (1:7 VN04) (low pathogenic)	Avian
H5N1, A/goose/Guiyang/337/2006 Clade 4 (7:1) (low pathogenic)	Avian
H5N1, A/chicken/Vietnam/NCVD-016/2008 Clade 7.1 (7:1) (low pathogenic)	Avian
**Influenza B Viruses**	
B/Yamagata/16/82 (Yamagata lineage)	Human
B/Texas/06/2011 (Yamagata Lineage)	Human
B/Nevada/03/2011 (Victoria Lineage)	Human
